# Inhibition of phosphatidylcholine-specific phospholipase C results in loss of mesenchymal traits in metastatic breast cancer cells

**DOI:** 10.1186/bcr3151

**Published:** 2012-03-19

**Authors:** Laura Abalsamo, Francesca Spadaro, Giuseppina Bozzuto, Luisa Paris, Serena Cecchetti, Luana Lugini, Egidio Iorio, Agnese Molinari, Carlo Ramoni, Franca Podo

**Affiliations:** 1 Department of Hematology, Oncology and Molecular Medicine, Istituto Superiore di Sanità, Viale Regina Elena 299, Roma, 00161, Italy; 2 Department of Cell Biology and Neurosciences, Istituto Superiore di Sanità, Viale Regina Elena 299, Roma, 00161, Italy; 3 Department of Technology and Health, Istituto Superiore di Sanità, Viale Regina Elena 299, Roma, 00161, Italy; 4 Department of Therapeutic Research and Medicines Evaluation, Istituto Superiore di Sanità, Viale Regina Elena 299, Roma, 00161, Italy

## Abstract

**Introduction:**

Acquisition of mesenchymal characteristics confers to breast cancer (BC) cells the capability of invading tissues different from primary tumor site, allowing cell migration and metastasis. Regulators of the mesenchymal-epithelial transition (MET) may represent targets for anticancer agents. Accruing evidence supports functional implications of choline phospholipid metabolism in oncogene-activated cell signaling and differentiation. We investigated the effects of D609, a xanthate inhibiting phosphatidylcholine-specific phospholipase C (PC-PLC) and sphingomyelin synthase (SMS), as a candidate regulator of cell differentiation and MET in the highly metastatic BC cell line MDA-MB-231.

**Methods:**

PC-PLC expression and activity were investigated using confocal laser scanning microscopy (CLSM), immunoblotting and enzymatic assay on human MDA-MB-231 compared with MCF-7 and SKBr3 BC cells and a nontumoral immortalized counterpart (MCF-10A). The effects of D609 on PC-PLC and SMS activity, loss of mesenchymal markers and changes in migration and invasion potential were monitored in MDA-MB-231 cells by enzymatic assays, CLSM, immunoblotting and transwell chamber invasion combined with scanning electron microscopy examinations. Cell proliferation, formation and composition of lipid bodies and cell morphology were investigated in D609-treated BC cells by cell count, CLSM, flow-cytometry of BODIPY-stained cells, nuclear magnetic resonance and thin-layer chromatography.

**Results:**

PC-PLC (but not phospholipase D) showed 2- to 6-fold activation in BC compared with nontumoral cells, the highest activity (up to 0.4 pmol/μg protein/min) being detected in the poorly-differentiated MDA-MB-231 cells. Exposure of the latter cells to D609 (50 μg/mL, 24-72 h) resulted into 60-80% PC-PLC inhibition, while SMS was transiently inhibited by a maximum of 21%. These features were associated with progressive decreases of mesenchymal traits such as vimentin and N-cadherin expression, reduced galectin-3 and milk fat globule EGF-factor 8 levels, β-casein formation and decreased *in vitro *cell migration and invasion. Moreover, proliferation arrest, changes in cell morphology and formation of cytosolic lipid bodies typical of cell differentiation were induced by D609 in all investigated BC cells.

**Conclusions:**

These results support a critical involvement of PC-PLC in controlling molecular pathways responsible for maintaining a mesenchymal-like phenotype in metastatic BC cells and suggests PC-PLC deactivation as a means to promote BC cell differentiation and possibly enhance the effectiveness of antitumor treatments.

## Introduction

Differentiation markers expressed by a primary breast cancer (BC) are currently profiled to guide prognosis and clinical decisions. Poorly differentiated tumors are held to be more aggressive and predictive of a less favorable response to treatment. There is increasing interest in regulators of the oncogenic epithelial-mesenchymal transition (EMT) and its reciprocal process, mesenchymal-epithelial transition (MET), for elucidation of the mechanisms underlying tumor progression and metastasis and the possible identification of new targets for cancer treatment [1].

The discovery of an abnormal choline phospholipid metabolism as the hallmark of BC and other cancers (reviewed in [2-5]) stimulated investigations on the possible role of phosphatidylcholine (PtdCho) cycle enzymes as potential indicators of tumor response and novel therapy targets [5-8]. Biochemical, genomic, and proteomic assays showed upregulation of choline kinase (ChoK) in BC and in epithelial ovarian cancer (EOC) cell lines [9-11]. RNA interference-mediated ChoK knockdown has been reported to exert anti-proliferative effects and induce cell differentiation in BC cells [12]. We recently showed potent increases of both ChoK and PtdCho-specific phospholipase C (PC-PLC) activities in EOC cells compared with non-tumoral counterparts [10,11]. PC-PLC isoforms responsible for PtdCho hydrolysis into phosphocholine and diacylglycerol (DAG) have been isolated but not yet cloned from mammalian sources. However, accruing evidence points to multiple implications of this enzyme in cell signaling through mitogen-activated protein kinase (MAPK) and oncogene-activated protein kinase pathways, programmed cell death, activation of immune cells, and stem cell differentiation ([13-19] and references therein). Furthermore, we reported direct evidence on PC-PLC activation and changes in subcellular localization of this enzyme in cancer [20,21] and non-tumoral receptor-activated mammalian cells [13,15-17]. In particular, selective PC-PLC accumulation was detected on the plasma membrane of EOC cells [20], human epidermal growth factor receptor 2 (HER2)-overexpressing BC cells [21], mitogen-stimulated fibroblasts [13], and cytokine-activated human natural killer cells [15-17]. The competitive PC-PLC inhibitor tricyclodecan-9-yl-potassium xanthate (D609) [22] used at the dose of 50 μg/mL (188 μM) blocked EOC cell proliferation [11] and prevented these cells from entering the S phase under growth factor stimulation [20]. Moreover, PC-PLC was found to associate with the HER2 receptor in raft domains of the plasma membrane of HER2-overexpressing BC cells [21]. In these cells, D609-induced PC-PLC inhibition resulted in HER2 receptor downregulation, together with that of its heterodimers with cognate members of the epidermal growth factor receptor family, by interfering with receptor internalization, degradation, and recycling. Overall, this body of evidence suggests the existence of regulatory links between PC-PLC activity, membrane receptor expression, and cancer cell proliferation.

On the other hand, at much higher doses (at least 500 μM), D609 not only inhibited cell proliferation but also reduced cell viability, eventually inducing apoptosis in the metastatic cell line MDA-MB-435 [23]. These effects were attributed to intracellular ceramide accumulation, as a result of D609-induced inhibition of sphingomyelin synthase (SMS) and activation of *de novo *ceramide synthesis.

In the present work, we report direct evidence of a sixfold constitutive PC-PLC upregulation in the poorly differentiated, highly metastatic BC cell line MDA-MB-231 compared with a non-tumoral counterpart, MCF-10A. Significant but lower increases in PC-PLC content and activity were also found in other BC cell lines (SKBr3 and MCF-7).

The rates of PC-PLC and SMS activity were measured in MDA-MB-231 cells in either the presence or absence of D609 (50 μg/mL). Special traits of MET and BC cell differentiation - such as decreased expression of vimentin and N-cadherin; downmodulation of molecules critically involved in tumor progression, such as galectin-3 and milk fat globule-epidermal growth factor 8 (MFG-E8); and production of β-casein - were detected in D609-treated MDA-MB-231 cells, together with long-standing and irreversible reduction of *in vitro *cell motility and invasion capabilities. Typical features of cell differentiation, such as proliferative arrest with maintenance of cell viability, changes in cell morphology, and formation of lipid bodies, were induced by D609 in all of the investigated BC cells.

## Materials and methods

### Cells

The human BC cell lines MDA-MB-231 (triple-negative: ER^-^, PgR^-^, and HER2^-^), SKBr3 (ER^-^, PgR^-^, and HER2^+^), and MCF-7 (ER^+^, PgR^+^, and HER2^-^) and the non-tumorigenic immortalized human mammary epithelial cell line MCF-10A were supplied by American Type Culture Collection (Manassas, VA, USA). The cells were cultured, as previously described [21], in either the presence or absence of D609 (Sigma-Aldrich, St. Louis, MO, USA).

### Antibodies and reagents

Rabbit polyclonal antibodies (Abs) raised against bacterial (*Bacillus cereus*) PC-PLC and selectively cross-reacting with mammalian PC-PLC were obtained in our laboratory [24] in accordance with a modification of the method originally described by Clark and colleagues [25] and characterized as reported [13,15]. Alexa Fluor**^®^-**633-conjugated phalloidin, 4,4-difluoro-1,3,5,7,8-pentamethyl-4-bora-3a, 4a-diaza-s-indacene (Bodipy 493/503), Bodipy-TR (BTR) ceramide, and the secondary Abs Alexa Fluor^®^-594 F(ab)_2 _fragments of goat anti-rabbit and goat anti-mouse IgG (H+L) were purchased from Molecular Probes Inc. (now part of Invitrogen Corporation, Carlsbad, CA, USA); mouse anti-β-actin and anti-vimentin Abs from Sigma-Aldrich; rabbit polyclonal anti-HER2, anti-E-cadherin, and anti-N-cadherin and mouse monoclonal anti-MFG-E8 from Santa Cruz Biotechnology, Inc. (Santa Cruz, CA, USA); monoclonal anti-galectin-3 and anti-β-casein Abs from Abcam (Cambridge, UK); and horseradish peroxidase-conjugated goat anti-mouse and goat anti-rabbit IgG from Bio-Rad Laboratories, Inc. (Hercules, CA, USA). Chemicals were from Sigma-Aldrich unless otherwise specified.

### Confocal laser scanning microscopy and flow cytometry analyses

For immunofluorescence analyses, cells were seeded in 24-well cluster plates onto 12-mm cover glasses. After 48 hours of culture in complete medium, cells were treated with or without D609 for different times, fixed in 3% paraformaldehyde (30 minutes at 4°C), permeabilized by Triton X-100 (0.5%, 10 minutes at room temperature), and then stained at 37°C with Bodipy 493/503, followed by Alexa Fluor^®^-633-conjugated phalloidin or by the primary and Alexa Fluor^®^-594-conjugated secondary Abs. The cover glasses were finally mounted on the microscope slide with Vectashield anti-fade mounting medium containing 4' 6-diamidino-2-phenylindole (DAPI) (Vector Laboratories, Burlingame, CA, USA). Confocal laser scanning microscopy (CLSM) observations were performed with a Leica TCS SP2 AOBS apparatus (Leica, Wetzlar, Germany), as described [20], by using excitation spectral laser lines at 405, 488, 594, and 633 nm. CLSM images were obtained by three-dimensional reconstruction of three or four optical sections.

For flow cytometry analyses, cells were detached from the substrate in phosphate-buffered saline ethylenediaminetetraacetic acid (PBS-EDTA) (5 mM). The fluorescence intensity of Bodipy 493/503 was measured on log-scale by using a FACScan (BD Biosciences, San Jose, CA, USA) apparatus. Apoptosis was evaluated by measuring the modulation of phosphatidylserine externalization by using Annexin V-biotin (Bender MedSystems GmbH, Vienna, Austria) followed by Alexa Fluor^®^-488-conjugated streptavidin (Molecular Probes Inc.). After treatment with D609 (50 μg/mL) for 24, 48, and 72 hours, cells were stained with Annexin V-biotin and 488-conjugated streptavidin and then analyzed by flow cytometry.

### Western blot analyses

According to our previously described procedure [20], protein expression was evaluated in total lysates (30 μg of proteins) from cells treated with or without D609 in complete medium.

### *In vitro *PC-PLC, phospholipase D, and sphingomyelin synthase activity assays

PC-PLC and phospholipase D (PLD) activity rates were determined in whole-cell lysates by using the Amplex Red assay kit (Molecular Probes Inc.) and a procedure described by the manufacturer and adapted by Spadaro and colleagues [16,20]. Changes of SMS activity were measured as described by Meng and colleagues [26] and adapted by Cecchetti and colleagues [17].

### Cell proliferation

MDA-MB-231, SKBr3, and MCF-7 cells were plated in six-well plates at a density of 1 × 10^5 ^cells per well for SKBr3 and 5 × 10^4 ^cells for MDA-MB-231 and MCF-7. After 48 hours of culture, cells were incubated with or without D609 for different time points. Afterwards, cells were detached from the substrate in PBS-EDTA (5 mM), and cell proliferation was evaluated by hemacytometer counting of viable Trypan blue-excluding cells.

### Nuclear magnetic resonance spectroscopy

Intact cells were counted, washed three times in PBS, centrifuged at 600*g*, and resuspended in PBS-D_2_O (700 μL) before transfer to 5-mm nuclear magnetic resonance (NMR) tubes (20 × 10^6 ^cells). ^1^H NMR analyses (25°C) were performed at 400 or 700 MHz (Bruker BioSpin Avance 400 or 700; Bruker BioSpin, Karlsruhe, Germany). Analyses of ^1^H NMR spectra and peak area deconvolution were performed as previously described [27].

### Lipid extraction and high-performance thin-layer chromatography analyses

Total lipid extracts obtained according to Folch and colleagues [28] were analyzed by thin-layer chromatography (TLC) by using cholesterol, cholesteryl esters, and triacylglycerols as standards [27]. Analyses were performed by staining the lipid bands with 2% copper acetate solution in 8% phosphoric acid and subsequent heating at 120°C for 15 minutes. The relative quantification of individual lipid classes was obtained by using the 'Quantity One' Bio-Rad software program (Bio-Rad Laboratories, Inc.) and normalized to the number of cells.

### Transwell chamber migration and invasion assays

The effects of D609 on the migration and invasive potentials of MDA-MB-231 cells were analyzed by a transwell chamber assay [29] by using inserts (8.0-μm pore, Falcon; BD, Franklin Lakes, NJ, USA) which stood in six-well plates (Costar, now part of Corning Life Sciences, Acton, MA, USA). In a first series of experiments, MDA-MB-231 cells were seeded in the transwell chambers either with or without D609 (50 μg/mL) and allowed to migrate to the lower side of the filter for 20 hours at 37°C. In a second series of experiments, MDA-MB-231 cells were first treated with or without D609 (50 μg/mL) for 24, 48, and 72 hours and subsequently detached and seeded in the transwell chambers for 20-hour assays in the absence of the inhibitor.

For the invasion assays, Matrigel™ (Sigma-Aldrich) was diluted to 1 mg/mL in serum-free Dulbecco's modified Eagle's medium (DMEM) and 250 μL was placed into the insert which stood in each well of the six-well plate. The inserts and the plate were incubated overnight at 4°C. The following day, cells were harvested and suspended in serum-free DMEM at a concentration of 1 × 10^6 ^cells per milliliter. The cell suspension was added to each insert, and 3 mL of DMEM containing 10% fetal bovine serum was added to the well underneath the insert. Cells were incubated at 37°C for 20 hours. After this time period, the inner side of the insert was wiped with a wet swab to remove the cells while the outer side of the insert was gently rinsed with PBS and stained with 0.25% crystal violet for 10 minutes, rinsed again, and then allowed to dry. The inserts were then viewed under the microscope. The detection of cells that had invaded through the membrane was performed under a computer-assisted color camera-equipped Nikon Optiphot microscope (Nikon Corporation, Tokyo, Japan), and the percentage of the area occupied by migrated cells (or their number) was analyzed by dedicated software (Optilab; Graftek Imaging Inc., Austin, TX, USA). The analysis was performed on 18 fields of each sample. The procedure for carrying out motility assays was identical to that used for invasion assays with the exception that inserts were not coated with Matrigel™.

### Scanning electron microscopy

Examinations were performed, as previously described [30], on a Cambridge Stereoscan 360 scanning electron microscope (Cambridge Instruments, Cambridge, UK).

### Statistical analysis

Data were analyzed by using GraphPad Software version 3.03 (GraphPad Software, Inc., La Jolla, CA, USA). Statistical significance of differences was determined by one-way analysis of variance (ANOVA) or by Student *t *test, as specified. Differences were considered significant at a *P *value of less than 0.05.

## Results

### PC-PLC overexpression and activation in MDA-MB-231 cells

**Figure 1:**
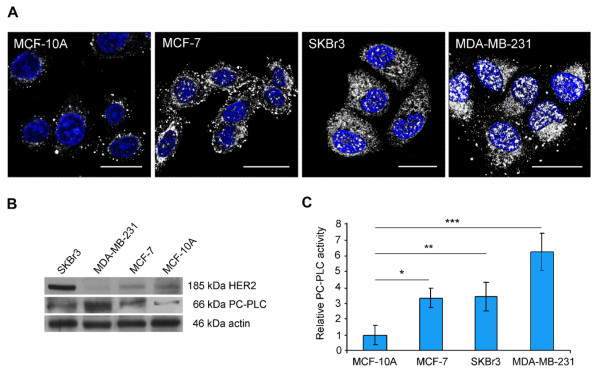
**Overexpression and activation of PC-PLC in human breast cancer cell lines**. **(a) **Confocal laser scanning microscopy examinations (three-dimensional reconstruction images) of fixed human breast cancer cells (MCF-7, SKBr3, and MDA-MB-231) and non-tumoral immortalized mammary epithelial cells (MCF-10A) that were stained for intracellular PC-PLC detection (pseudo-color gray). Nuclei were stained with DAPI (blue). Scale bars, 20 μm. **(b) **Representative Western blot analyses (of three independent experiments) of cell lysates show PC-PLC and HER2 protein expression (actin was used as a quantitative loading control). **(c) **Relative PC-PLC activity (presented as mean ± standard deviation, n = 3) in cancer versus non-tumoral cells at early confluence. Student *t *test, *P = 0.007, **P < 0.003, ***P < 0.0001; one-way analysis of variance of tumoral versus non-tumoral cells, *P *< 0.0001. DAPI, 4',6-diamidino-2-phenylindole; HER2, human epidermal growth factor receptor 2; PC-PLC, phosphatidylcholine-specific phospholipase C.

Differential PC-PLC expression and activity were measured in MDA-MB-231 cells and compared with those of the other investigated BC cells and the non-tumoral counterpart by using CLSM analyses, Western blot, and biochemical assays. Figure 1a shows the intracellular distribution of PC-PLC in fixed and permeabilized cells, stained with the anti-PC-PLC Ab. The highly metastatic MDA-MB-231 cell line showed the highest PC-PLC content, distributed in both nuclear and cytoplasmic compartments, including the inner filamentous structures directed from perinuclear area to the cell periphery. A qualitatively similar intracellular PC-PLC distribution was exhibited by SKBr3 and MCF-7 cell lines in which, however, the overall PC-PLC content appeared to be lower than that of MDA-MB-231 cells. Only a few PC-PLC-positive granules were instead detected in MCF-10A cells, where they were concentrated mainly in perinuclear areas and were practically absent in intranuclear regions.

Western blot analyses of total cell lysates (Figure 1b) allowed detection of a PC-PLC isoform with an apparent molecular weight (M_r_) of 66 kDa, which is in agreement with previous studies by our group and other groups on a number of different mammalian systems [13,15-18,20,21,24,25,31]. Densitometric analyses confirmed that the MDA-MB-231 cells expressed the highest PC-PLC content, and the factor of increase was 6.0 ± 1.6 (± standard deviation) in comparison with the non-tumoral counterpart. All BC cells showed a higher PC-PLC protein expression in comparison with MCF-10A cells (one-way ANOVA *P *< 0.002), but the factors of increase were lower in SKBr3 (4.9 ± 0.9) and MCF-7 (3.4 ± 0.7) than in MDA-MB-231 cells. As shown in Figure 1c, Amplex Red assays on total lysates from cells harvested at early confluence also showed a 6.3 ± 1.2-fold increase in the PC-PLC activity in MDA-MB-231 cells in comparison with the non-tumoral counterpart, whereas the factors of increase were lower for the other BC cells (3.4 ± 0.9 for SKBr3 and 3.3 ± 0.6 for MCF-7). By contrast, the PLD activity was not significantly different among BC and non-tumoral cells (Additional file 1). Altogether, these results showed that the highest PC-PLC upregulation occurred in the poorly differentiated MDA-MB-231 cells.

### Cell proliferation arrest in MDA-MB-231 cells exposed to D609

**Figure 2:**
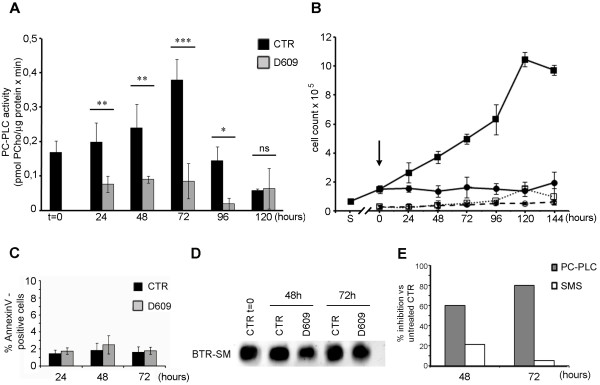
**PC-PLC and SMS activity inhibition and cell growth arrest in MDA-MB-231 cells exposed to D609 (50 μg/mL)**. **(a) **Absolute PC-PLC activity (mean ± SD, n = 4) measured in total lysates of MDA-MB-231 cells harvested after exposure to D609 (gray columns) in comparison with untreated cells (CTR, black columns). Student *t *test, *P ≤ 0.04, **P ≤ 0.02, ***P = 0.0002; ns, not significant. **(b) **Cell counts (mean ± SD, n = 3) of MDA-MB-231 cells incubated in the absence (black square, viable cells; white square, dead cells) or presence (black circle, viable cells; white circle, dead cells) of D609. Cells were seeded (S) 48 hours before treatment, which started at t = 0 (arrow). **(c) **Percentage of Annexin V-positive cells in D609-treated and the respective CTR cells. **(d) **Relative SMS activity detected in MDA-MB-231 cells incubated in the absence (CTR) or presence of D609 for the indicated times. Lipid extracts were added with BTR-ceramide, and the formation of the SMS product, BTR-sphingomyelin (BTR-SM), was detected by thin-layer chromatography and quantified by densitometric analysis (representative example of two independent experiments). **(e) **Comparison of the percentages of PC-PLC (gray) and SMS (white) inhibition induced in MDA-MB-231 cells by exposure to D609 for 48 or 72 hours. BTR, Bodipy-TR; PCho, phosphocholine; PC-PLC, phosphatidylcholine-specific phospholipase C; SD, standard deviation; SMS, sphingomyelin synthase.

The absolute PC-PLC activity of untreated (control, or CTR) MDA-MB-231 cells increased in the log-phase of growth from 0.2 to 0.4 pmol/μg protein per minute between 24 and 72 hours (early confluence) and decreased thereafter (Figure 2a). Cell exposure to D609 (50 μg/mL, 188 μM) inhibited the PC-PLC activity by 60% at 24 to 48 hours and by 80% at 72 hours.

Continuous exposure of MDA-MB-231 cells to this dose of D609 induced a long-standing cell proliferation arrest up to at least 144 hours (Figure 2b). Similar anti-proliferative effects were found for D609-treated SKBr3 and MCF-7 cells (Additional file 2, panels a and b). The D609-induced inhibition of cancer cell growth was not due to general cytotoxicity, because the number of dead cells was practically maintained at the same levels in BC and in their control cultures (Figure 2b and Additional file 2a and b). The difference in the percentage of dead cells in untreated compared with treated BC cell cultures was therefore due to D609-induced inhibition of cell proliferation rather than to an increase in cell mortality. Moreover, measurement of the percentages of Annexin V-positive cells showed that, at this dose, D609 did not exert any substantial apoptotic effect on any of the investigated BC cells (Figure 2c and Additional file 2c and d).

A massive loss of cell viability was instead detected in MDA-MB-231 cell cultures exposed to much higher D609 doses (at least 500 μM), as shown in panels a and b of Additional file 3. In cells treated for 48 hours, the percentage of dead cells increased from 12.5% ± 4.5% at the dose of 188 μM to 69.3% ± 14.1% at 500 μM and 88.9% ± 8.1% at 750 μM, compared with 5.1% ± 2.7% in control cells. Similar differential levels were detected at 72 hours. At the dose of D609 henceforth used throughout this study (50 μg/mL), the SMS activity was inhibited by only 21% at 48 hours and 5% at 72 hours (Figure 2d). Therefore, the inhibition of SMS, compared with that of PC-PLC, was threefold lower at 48 hours and 16-fold lower at 72 hours (Figure 2e). Overall, these results showed that, at the dose of 50 μg/mL, the most relevant inhibitory effect of D609 on MDA-MB-231 cells was targeted against PC-PLC.

### Formation of cytoplasmic lipid bodies and changes of cell morphology in D609-treated MDA-MB-231 cells

**Figure 3:**
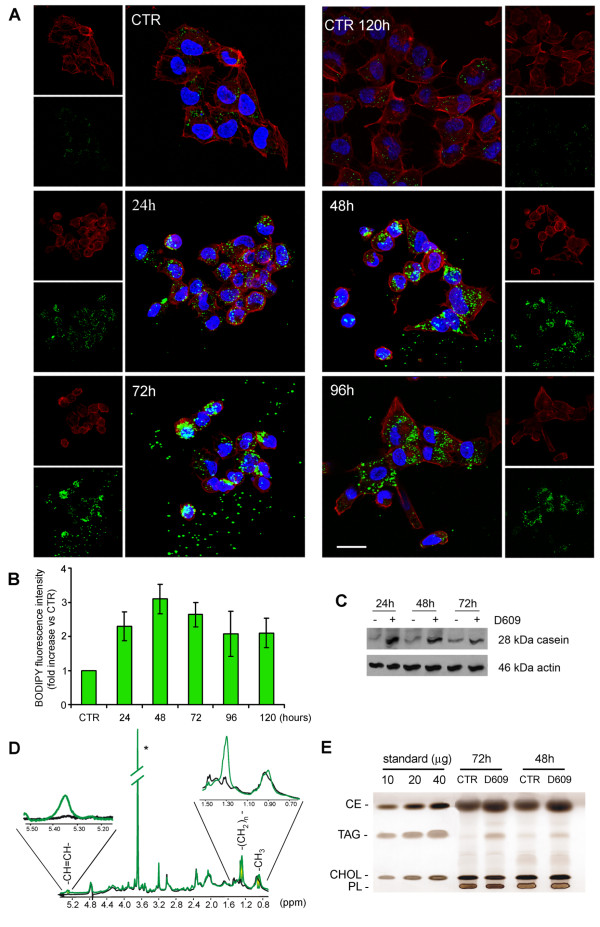
**Formation of cytoplasmic lipid bodies and production of β-casein in MDA-MB-231 cells exposed to D609**. **(a) **Confocal laser scanning microscopy analyses (three-dimensional reconstruction images) of MDA-MB-231 cells cultured in the absence (CTR) or presence of D609 (50 μg/mL) for the indicated times and stained with Bodipy 493/503 (green). Morphological changes of actin cytoskeleton were monitored by phalloidin-633 staining (red); nuclei are reported in blue (DAPI). Scale bar, 20 μm. **(b) **Histogram showing the fold increase in Bodipy 493/503 fluorescence intensity measured by flow cytometry in D609-treated MDA-MB-231 cells in comparison with CTR (mean ± standard deviation, n = 3). **(c) **Changes in β-casein expression detected by Western blot in MDA-MB-231 cells after incubation with D609 for the indicated times (actin as loading control). **(d) **Representative proton nuclear magnetic resonance (^1^H NMR) spectrum of intact MDA-MB-231 cells incubated for 48 hours in the absence (black) or presence (green) of D609, showing increases in -(CH_2_)_n_- (1.30 ppm) and = CH-CH = (5.34 ppm) signals of mobile lipids in D609-treated cells (details in inserts). Asterisk indicates unidentified signal of commercial phosphate-buffered saline buffer. **(e) **Thin-layer chromatography analysis of total lipid extracts of D609-treated MDA-MB-231 cells in comparison with CTR. Fold increases were as follows: triacylglycerols (TAG), 1.8 ± 0.1 at 48 hours and 1.8 ± 0.8 at 72 hours (n = 3); cholesteryl esters (CE), 1.4 ± 0.1 at 48 hours and 1.7 ± 0.5 at 72 hours; cholesterol (CHOL), 1.0 ± 0.1 at 48 hours and 1.1 ± 0.1 at 72 hours. Phospholipids (PL) were not significantly altered. CTR, control; DAPI, 4',6-diamidino-2-phenylindole; ppm, parts per million.

The maturation of breast cells is typically characterized by the formation of cytoplasmic lipid bodies and production of the milk protein β-casein [32]. CLSM analyses showed that only a few lipid vacuoles were present in MDA-MB-231 cells (Figure 3a) cultured in complete medium (CTR) and stained with Bodipy 493/503 (green), a fluorescent hydrophobic molecule that selectively localizes to neutral lipid aggregates [33]. However, when these cells were incubated with D609 (50 μg/mL), lipid bodies were already detected at 24 hours and their number increased at 48 to 72 hours and remained at high levels thereafter. Furthermore, during D609 incubation, cells progressively underwent morphological changes by retracting the cytoplasm toward the nucleus (rounded morphology at 24 to 72 hours) and displaying a flattened morphology with expansion of the cytoplasm at longer times, a characteristic feature of mature breast cells [32,34]. Flow cytometry analyses of Bodipy-stained cells showed up to threefold to fourfold increases in the mean fluorescence intensity of D609-treated MDA-MB-231 cells in comparison with the untreated control, and the maximum was at 48 to 72 hours (Figure 3b). Similar morphological changes and induction of lipid bodies were observed in D609-treated SKBr3 and MCF-7 cells (Additional files 4 and 5). Western blot analyses showed formation of β-casein, which already occurred in MDA-MB-231 cells at 24 hours of exposure to D609 (Figure 3c).

The intracellular formation of isotropically tumbling lipid bodies was confirmed by ^1^H NMR spectra of intact MDA-MB-231 cells incubated for 48 hours with D609 (Figure 3d), in which a fourfold increase was measured in the area of the resonance at 1.30 parts per million (ppm), typical of saturated -(CH_2_)_n_- segments of mobile lipid fatty acyl chains ([27] and references therein). Moreover, a clear-cut increase of the -CH = CH- resonance (at 5.34 ppm) indicated that these chains were partially unsaturated.

TLC analyses of lipid extracts showed an average 1.8-fold increase in triacylglycerols and 1.4- to 1.7-fold increases in cholesteryl esters at 48 to 72 hours of cell exposure to D609, whereas cholesterol and the overall phospholipid contents remained unaltered (Figure 3e). Overall, these experiments showed that exposure to D609 induced the following in the metastatic MDA-MB-231 cells: intracellular accumulation of cytoplasmic lipid bodies, expression of β-casein, and morphological changes typical of breast cell maturation.

### Decrease of mesenchymal traits and markers of tumorigenesis in D609-treated MDA-MB-231 cells

**Figure 4:**
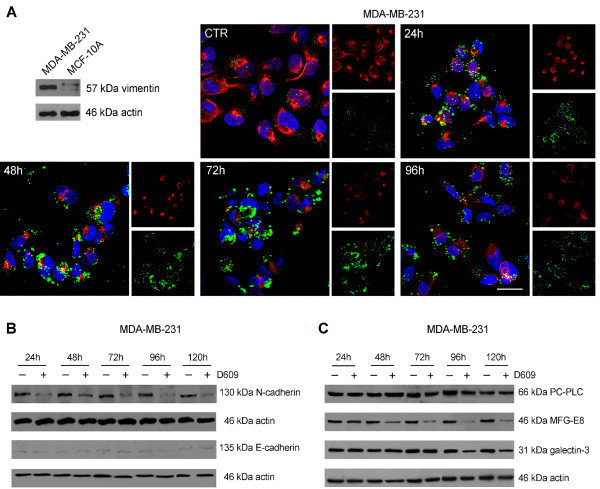
**Effects of D609 on the expression of vimentin and other differentiation markers in MDA-MB-231 cells**. **(a) **Vimentin expression detected by Western blot in MDA-MB-231 and MCF-10A cells (actin, loading control). The expression and distribution of vimentin (red) were evaluated by confocal laser scanning microscopy (three-dimensional reconstruction images) in D609-treated or untreated (CTR) MDA-MB-231 cells. Lipid body formation was monitored by Bodipy 493/503 staining (green). Nuclei are reported in blue (DAPI). Scale bar, 20 μm. **(b, c) **Changes in protein expression of N-cadherin, E-cadherin, PC-PLC, MFG-E8, and galectin-3, evaluated by Western blot analyses of MDA-MB-231 cells incubated with D609 (actin, loading control). Experiments in panels (a-c) were independently repeated three times with similar results. CTR, control; DAPI, 4',6-diamidino-2-phenylindole; PC-PLC, phosphatidylcholine-specific phospholipase C.

A typical feature of the mesenchymal phenotype is the overexpression of vimentin, an intermediate filament associated with increased invasive and metastatic potential of BC cells [35]. As shown in Figure 4a, vimentin expression was high in MDA-MB-231 cells but was barely detectable in MCF-10A cells. A progressive decrease of vimentin was detected in MDA-MB-231 cells, starting from 24 hours of exposure to D609, and 33% ± 4% of cells became vimentin-negative at 96 hours (Figure 4a) and 50% ± 17% at 144 hours (not shown). The simultaneous formation of cytoplasmic lipid bodies was confirmed by Bodipy staining (Figure 4a, green).

Partial reversal of the mesenchymal-like phenotype in D609-treated MDA-MB-231 cells was further supported by a strong decrease of N-cadherin (by 74% ± 4% at 24 hours, Student *t *test P = 0.01, and 75% ± 9% at 120 hours, *P *≤ 0.005), whereas E-cadherin maintained practically undetectable levels throughout cell incubation with D609 (Figure 4b).

Exposure of MDA-MB-231 cells to D609 also resulted in decreased galectin-3 (Figure 4c), a protein implicated in cancer cell growth, adhesion, angiogenesis, and metastatic potential [36,37]. The reduction in galectin-3 expression became substantial only at long times of D609 exposure, and decreases of 51% ± 13% at 96 hours and 65% ± 16% at 120 hours were observed. Lastly, a substantial reduction in the expression of MFG-E8, reputed to be a promoter of tumorigenesis in triple-negative BC [38,39], was detected in D609-treated MDA-MB-231 cells, and average decreases of 61% ± 3% at 48 hours and 83% ± 4% at 120 hours were observed (Figure 4c).

Unlike the content of MFG-E8 and galectin-3, that of PC-PLC was maintained substantially unaltered in MDA-MB-231 cells exposed to D609. Independent Western blot experiments, performed by using glyceraldehyde-3-phosphate dehydrogenase (GAPDH) as a loading control, showed that the actin level was also kept unmodified (data not shown). Overall, these results support the view that D609-induced PC-PLC inhibition was associated in MDA-MB-231 cells with the loss of some markers typical of mesenchymal phenotype and tumorigenesis.

### Decrease of migration and invasion potential in D609-treated MDA-MB-231 cells

**Figure 5:**
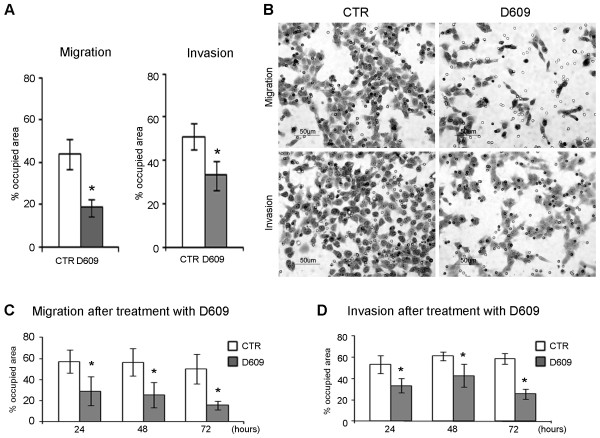
**Quantitative analysis of the migration and invasion potential of MDA-MB-231 cells**. The analysis was performed after staining with crystal violet the transwell chamber inserts to detect the cells migrated to the lower side of the porous membranes in the absence (migration) or presence (invasion) of Matrigel™. White columns represent control cells, and gray columns represent D609-treated cells. **(a) **Percentage of area occupied by migrating and invading cells in the absence or presence of D609. **(b) **Representative membranes containing cells that migrated in the absence or presence of Matrigel™. Examples of migration **(c) **and invasion **(d) **of MDA-MB-231 cells that were first treated with D609 (50 μg/mL) for 24, 48, and 72 hours, were subsequently detached, and were finally seeded in the transwell chambers and allowed to migrate in the absence of the inhibitor. CTR, control.

The quantitative analysis of migration and invasion potential was performed on membranes stained with crystal violet, as described in Materials and methods. The analyses were carried out by estimating either the percentage of area occupied by the cells (Figure 5) or the number of cells that migrated to the lower side of the filter (Additional file 6).

**Figure 6:**
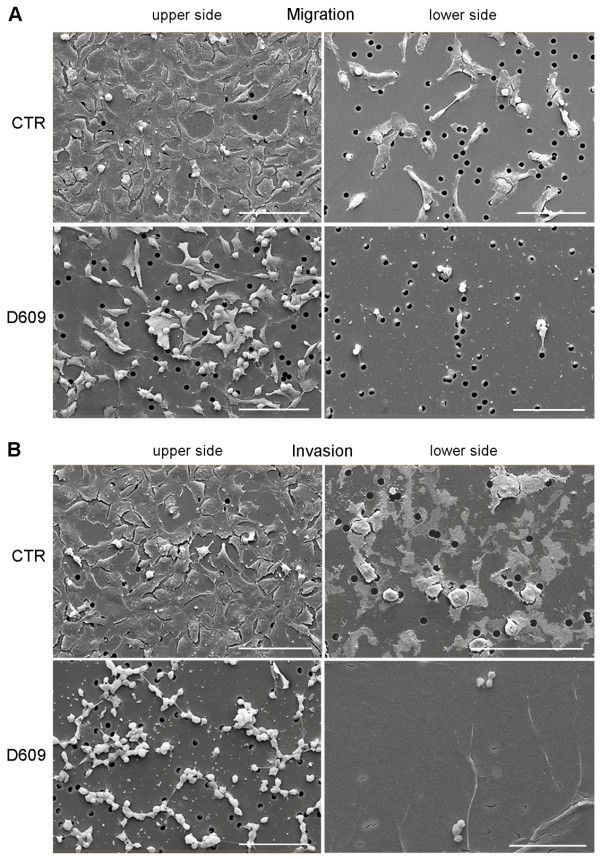
**Qualitative analysis of the migration and invasion potential of MDA-MB-231 cells**. Scanning electron microscopy observations were performed at the end of the 20-hour assays in the absence (migration) **(a) **or presence of Matrigel™ (invasion) **(b) **on cells treated with or without D609 (50 μg/mL). The imaging was performed on both the upper and lower sides of the filter. On the upper side, adherent cells are visible. On the lower side, cells that migrated through the pores of the membrane are depicted. Scale bars, 100 μm. CTR, control.

In the first series of experiments described in Materials and methods, cells were seeded in transwell chambers and allowed to migrate across the filter or invade the Matrigel™ for 20 hours, either with or without D609 (50 μg/mL). Quantitative analyses showed that the presence of D609 significantly inhibited both cell motility and invasion (see histograms in Figure 5a and Additional file 6a). Qualitative examinations by scanning electron microscopy showed that the migrating or invading untreated cells (CTR) adopted a polygonal and flat morphology when they adhered to the upper side of the filter and moved individually across the pores in either the absence (Figure 6a) or presence (Figure 6b) of Matrigel™. Exposure to D609 induced morphological changes on the migrating cells, which frequently appeared less flattened and even roundish (Figure 6a). In invasion assays, D609-treated cells showed a markedly round morphology and clustered together (Figure 6b). These features are known to reflect the reorganization of actin microfilaments in viable migrating or invading cells, as demonstrated by previous fluorescence microscopy studies (for example, [40]). As shown in Figure 6b, in the presence of D609, very few migrating cells were observed on the lower side of the filter. Matrigel™ film, in fact, appeared intact, suggesting that D609 inhibited both the cell movement and the matrix proteolysis.

In the second series of experiments, cells were separately incubated with D609 for 24, 48, and 72 hours, washed, and then transferred to the transwell chambers in the absence of D609. Significantly reduced migration and invasion capabilities were confirmed for the D609-treated cells in comparison with untreated controls (Figure 5c and 5d and Additional file 6c and d), providing evidence that these effects were not reverted during the 20-hour migration and invasion assays performed in the absence of the inhibitor.

## Discussion

This study reports the first evidence of a high (sixfold) overexpression and activation of PC-PLC (but not PLD) in a highly metastatic, triple-negative BC cell line (MDA-MB-231) in comparison with a non-tumoral counterpart. Substantial, though lower, upregulation of PC-PLC was also detected in the luminal-like MCF-7 and in the HER2-positive SKBr3 cell line.

A strong (60% to 80%) PC-PLC inhibition was induced in MDA-MB-231 cells by 24- to 72-hour exposure to D609 at the dose of 50 μg/mL. Under these conditions, these and other BC cells underwent proliferation arrest in the absence of apoptosis, along with morphological changes typical of cell differentiation.

**Figure 7:**
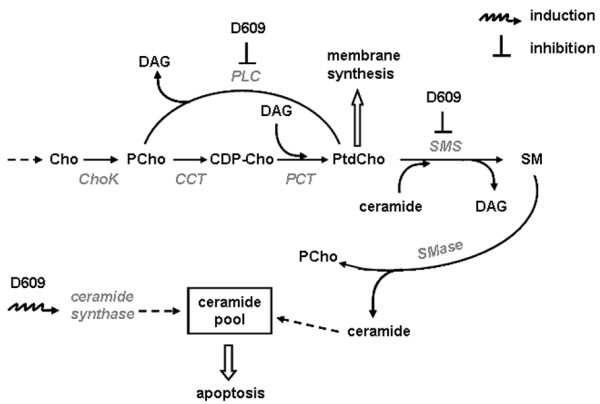
**Schematic representation of major links between pathways of biosynthesis and catabolism of phosphatidylcholine (PtdCho) and sphingomyelin (SM) and their relationships with membrane synthesis and apoptosis**. The dose-dependent effects of D609 on phospholipase C (PLC) and sphingomyelin synthase (SMS) inhibition and on stimulation of ceramide synthase are described in the Discussion section. CCT, phosphocholine-cytidylyl transferase; CDP-Cho, cytidine 5'-diphosphocholine; Cho, free choline; ChoK, choline kinase; DAG, diacylglycerol; PCho, phosphocholine; SMase, sphingomyelinase.

Figure 7 shows some basic links between pathways of biosynthesis and catabolism of PtdCho and sphingomyelin, together with their relations with two major biological effects: membrane synthesis and apoptosis. At the D609 dose used in our study, inhibition of SMS was 3- to 16-fold lower than that of PC-PLC at 48 to 72 hours of cell exposure to this agent. At doses that were 2.5- to 5.3-fold higher, D609 has been reported to induce apoptosis in the highly metastatic MDA-MB-435 carcinoma cell line, likely because of activation of ceramide synthase and stronger SMS inhibition with consequent accumulation of ceramides [23]. A massive loss of cell viability was also detected in our study in BC cell cultures of different subtypes exposed to similarly high doses of D609.

In regard to possible effects exerted by D609 on the activity of other enzymes, previous analyses of reaction mixtures showed that D609 did not directly inhibit PLD, phosphatidylinositol-specific phospholipase C (PI-PLC), phospholipase A2 [22], or sphingomyelinase [24]. However, an increase, rather than inhibition, of PLD-mediated PtdCho hydrolysis has been reported in lysates of osteoblastic osteosarcoma cells exposed to D609 at the dose of 50 μg/mL [41]. This effect, possibly due to mechanisms taking place in the cell to compensate for PC-PLC inhibition, was not associated with changes in the cell differentiation status.

The major finding of this study was that the strong inhibitory effect exerted by a non-apoptotic dose of D609 on PC-PLC activity in MDA-MB-231 cells was associated with the acquisition by these cells of a series of features typical of the MET process and cell differentiation, such as (a) progressive depletion of vimentin and N-cadherin expression, (b) significant reduction of *in vitro *cell migratory and invasive capabilities, (c) formation of cytoplasmic lipid bodies and production of the milk protein β-casein, and (d) decrease in the levels of two markers of cancer cell aggressiveness: MFG-E8 and galectin-3.

These results and their interpretation also provided a reason to further investigate the reversibility of the effects induced by the PC-PLC inhibitor on BC cell differentiation. Our study showed that, although the D609-induced MET was not complete (E-cadherin was not formed), some of the effects induced by this agent, such as reduced migration and invasion capabilities, were not reverted when D609 was withdrawn from the medium.

This body of evidence supports the views that (a) a high PC-PLC activity is associated with a poorly differentiated BC cell phenotype and (b) PC-PLC inhibition likely contributes to the molecular mechanisms leading these cells across a partial MET and cell differentiation.

### PC-PLC activity as a possible mechanistic regulator of EMT/MET switch in metastatic breast cancer cells

EMT is a major multistep process in BC progression, comprising the acquisition of mesenchymal features associated with dissolution of the epithelial integrity, cell proliferation, increased migration and local invasion, and, ultimately, distant metastasis [35,42-44]. Less-differentiated stem-like properties typical of the mesenchymal status [45] are reported for highly malignant BC cells which, compared with epithelial cells, commonly present higher vimentin and N-cadherin and low, if any, E-cadherin expression [35]. These molecular events lead to a less rigid cytoskeleton, reduced cell-cell contact, acquisition of cell-elongated shape, cell invasiveness, and metastasis. Our study shows that a substantial portion of these features were lost in MDA-MB-231 cells in which continuous exposure to D609 induced a strong and persistent PC-PLC inhibition. Although vimentin and N-cadherin losses were not associated with any rise in E-cadherin expression, a late marker of the MET process, it is worth noting that other characteristic features of BC cell differentiation (cell growth arrest, formation of intracellular lipid bodies, and β-casein production) were distinctly detected during D609 treatment.

The high level of MFG-E8 detected in the metastatic MDA-MB-231 cells is in agreement with a recent report showing that this α_v_β_3-5 _integrin ligand is a potential metastasis-associated tumor biomarker of triple-negative BC cells [39]. The decrease in MFG-E8 expression in D609-treated MDA-MB-231 cells, reported here, deserves further investigations in light of an increased sensitivity to cisplatin reported for triple-negative BC cells following p63 and MFG-E8 knockdown by siRNA (short interfering RNA) transfection [39].

Additional support for a possible role of PC-PLC inhibition in enhancing the sensitivity of metastatic BC cells to drug-induced cytotoxicity may be provided by the decrease of galectin-3 in D609-treated MDA-MB-231 cells, also reported here. In fact, inhibition of galectin-3 by a synthetic agent was recently reported to increase the sensitivity of a pulmonary BC metastasis to taxol-induced apoptosis *in vitro *and *in vivo *[46].

### Possible molecular mechanisms sustaining the role of PC-PLC activity as a regulator of breast cancer cell differentiation

Although the molecular bases of EMT and MET have not been fully elucidated, inter-linked transduction pathways and signaling molecules, including growth factors, tyrosine kinase receptors, and Ras effector-activated MAPK and phoshoinositide 3 kinase/AKT/mammalian target of rapamycin (PI3K/AKT/mTOR) axes, are reputed to be involved in key processes such as control of cell proliferation, shape remodeling, motility, and metastasis [47,48]. The strong activation of PC-PLC in the highly metastatic MDA-MB-231 cells, reported here, and the loss of mesenchymal traits crucial to cytoskeletal reorganization, cell motility, and invasion in BC cells exposed to a PC-PLC inhibitor suggest that the PC-PLC activity status may play a pivotal role in the EMT/MET switch.

**Figure 8:**
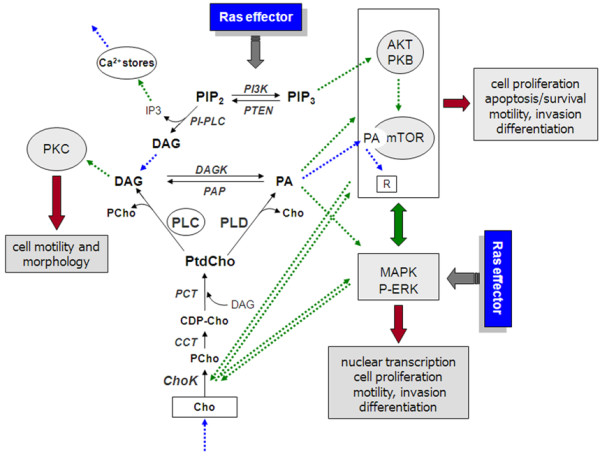
**Proposed role of PC-PLC in piloting cell signaling effects on breast cancer cell proliferation and differentiation**. Metabolites/second messengers: CDP-Cho, cytidine 5'-diphosphocholine; Cho, free choline; DAG, diacylglycerol; IP3, inositol (1,4,5)-trisphosphate; PA, phosphatidate; PCho, phosphocholine; PIP2, phosphatidylinositol (4,5)-bisphosphate; PIP3, phosphatidylinositol (3,4,5)-trisphosphate; PtdCho, phosphatidylcholine. Inhibitors: R, rapamycin and its analogs. Enzymes/protein kinases: AKT/PKB, AKT/protein kinase B; CCT, phosphocholine-cytidylyl transferase; CDP-Cho, cytidine 5'-diphosphocholine; ChoK, choline kinase; DAGK, diacylglycerol kinase; MAPK, mitogen-activated protein kinase; mTOR, mammalian target of rapamycin; PAP, phosphatidate phosphohydrolase; PC-PLC, phosphatidylcholine-specific phospholipase C; PCT, phosphocholine diacylglycerol transferase; P-ERK, phosphorylated extracellular signal-regulated kinase; PI3K, phosphoinositide 3-kinase; PI-PLC, phosphoinositide-specific phospholipase C; PKC, protein kinase C; PLD, phospholipase D; PTEN, phosphatase and tensin homolog. Black arrows indicate chemical reactions, dotted green arrows represent stimulation or other link, blue dotted arrows represent molecular transport or transfer to a different site, and solid gray arrows point to biological effects.

As schematically represented in Figure 8, PC-PLC works at the crossroad of major cell signaling pathways responsible for cell proliferation, motility, and differentiation. In fact, a PC-PLC-mediated DAG release from PtdCho may contribute to a long-lasting activation of protein kinase C (PKC), a family of isoenzymes involved in different functions, including regulation of BC cell morphology, motility, and invasiveness [49]. A decrease in the DAG pool as a result of PC-PLC inhibition could therefore lead to reduced cell motility due to partial PKC deactivation and subsequent cytoskeletal rearrangements at the cell-leading edge, similarly to the effects of DAG depletion detected in cancer cells exposed to PI-PLC-γ inhibitors [50].

Furthermore, a switch in the PC-PLC activation status could interfere with the biological effects of the two inter-linked MAPK and PI3K/AKT/mTOR axes. The PC-PLC-mediated DAG production can, in fact, be partly converted by DAG kinase (DAGK) into phosphatidate, a potent mitogen reported to stimulate MAPK and to act as an antagonist of rapamycin at the mTORC-1 complex-binding site [51-53]. PC-PLC-driven changes in the phosphatidate content can, therefore, be expected to influence (a) the proliferative/anti-proliferative effects exerted by these signaling pathways, (b) the migratory/anti-migratory effects exerted by rapamycin-sensitive downhill targets of mTOR at the level of the G_1_-to-S transition and cell motility [48], and (c) the balance of anti-apoptotic effects exerted by antagonists of cell death.

## Conclusions

The results reported here support the view that a PC-PLC activation/deactivation switch may act as a regulator of molecular mechanisms responsible for redirecting EMT to MET and inducing cell differentiation in BC cells. This hypothesis suggests the possible use of PC-PLC as a new target for anti-cancer therapy, which may leave non-neoplastic tissues unaffected. Preclinical *in vivo *investigations to evaluate the role of PC-PLC inhibitors to enhance the effectiveness of therapies against poorly differentiated BCs, including triple-negative BCs, are, therefore, warranted.

## Abbreviations

Ab: antibody; ANOVA: analysis of variance; BC: breast cancer; ChoK: choline kinase; CLSM: confocal laser scanning microscopy; CTR: control; DAG: diacylglycerol; DMEM: Dulbecco's modified Eagle's medium; EMT: epithelial-mesenchymal transition; EOC: epithelial ovarian cancer; ER: estrogen receptor; ^1^H NMR: proton nuclear magnetic resonance; HER2: human epidermal growth factor receptor 2; MAPK: mitogen-activated protein kinase; MET: mesenchymal-epithelial transition; MFG-E8: milk fat globule-epidermal growth factor 8; mTOR: mammalian target of rapamycin; NMR: nuclear magnetic resonance; PBS: phosphate-buffered saline; PBS-EDTA: phosphate-buffered saline ethylenediaminetetraacetic acid; PC-PLC: phosphatidylcholine-specific phospholipase C; PgR: progesterone receptor; PI3K: phoshoinositide 3 kinase; PI-PLC: phosphatidyl inositol-specific phospholipase C; PKC: protein kinase C; PLD: phospholipase D; ppm: parts per million; PtdCho: phosphatidylcholine; SMS: sphingomyelin synthase; TLC: thin-layer chromatography.

## Competing interests

The authors declare that they have no competing interests.

## Authors' contributions

LA and FS carried out the CLSM, PC-PLC enzymatic assays, and immunoassays and drafted the manuscript. LP and SC performed the acquisition and analysis of the flow cytometry data and participated in the immunoassays. LL tested cell proliferation and carried out the SMS assays in collaboration with LP. EI performed NMR spectroscopy, TLC, and statistical analyses. GB and AM carried out the transwell chamber invasion assays and scanning electron microscopy examinations. CR participated in the design of the study and in the interpretation of data. FP conceived the study, participated in its design and discussion of the results, and helped to draft the manuscript. All authors read and approved the final manuscript.

## Authors' information

LA presented this work as her thesis for a degree in biological sciences at the University of Rome 'La Sapienza'. She recently obtained her PhD and is a research fellow at the Istituto Superiore di Sanità (ISS) in Rome. FS, LP, SC, and LL are PhD researchers at the ISS. GB is a PhD student and research fellow at the ISS. EI is a researcher at the ISS. AM is the director of the Ultrastructural Methods for Innovative Anticancer Therapies Unit at the Department of Technology and Health of the ISS. CR was senior investigator and FP, research director, acted as head of the Molecular and Cellular Imaging Unit at the Department of Cell Biology and Neurosciences of the ISS; she is currently a fellow of the Italian Association Alleanza Contro il Cancro (ACC) in the Department of Hematology, Oncology, and Molecular Medicine of the ISS.

## Supplementary Material

Additional file 1 **Relative PC-specific phospholipase D (PLD) activity in breast cancer cells**. PLD activity was measured by Amplex Red assay in breast cancer (BC) cell lines (MCF-7, SKBr3 and MDA-MB-231) compared with the human nontumoral mammary epithelial cells MCF-10A, all harvested at early confluence. Relative fold changes in BC cell PLD activity were normalized to the activity of MCF-10A cells, set to 1. Histograms represent the mean ± SD (n = 3). No significant differences were found in the relative rates of PLD in all investigated cell lines. Click here for file

Additional file 2 **Proliferation arrest induced by D609 (50 μm/mL) in SKBr3 and MCF-7 cells**. **(A) **and **(B) **Cell counts (mean ± SD, n = 3) of SKBr3 and MCF-7 cells incubated in absence (black square, viable cells; white square, dead cells) or presence of D609 (black circle, viable cells; white circle, dead cells). Cells were seeded (S) 48 h before treatment, which started at t = 0 (arrow). **(C) **and **(D) **Percentages of Annexin V-positive cells in D609-treated and the respective CTR cells. Click here for file

Additional file 3 **Effects of different doses of D609 on cell growth and viability of MDA-MB-231 cells**. Cells were incubated with different doses of D609 (188 μM, (corresponding to 50 μg/mL), 500 μM and 750 μM) for 48 h or 72 h. Cell proliferation was measured by cell count. Cell viability was assessed by trypan blue excluding test (experiments performed in triplicate). Click here for file

Additional file 4 **Induction of intracellular lipid bodies in SKBr3 cells following exposure to the PC-PLC inhibitor D609**. CLSM analyses (three-dimensional reconstruction images) of cells exposed to D609 (50 μg/mL) for the indicated time intervals, then fixed and stained with the lipid probe BODIPY 493/503 (green) for the detection of cytoplasmic lipid bodies and with phalloidin-633 (red) for monitoring morphological changes of actin cytoskeleton. Nuclei are reported in blue (DAPI). The corresponding control cell cultures are reported in the panels indicated by 'CTR' and 'CTR 72 h'. Scale bar, 20 μm. *Histogram on the bottom panel*: fold-increase of BODIPY 493/503 fluorescence intensity measured by flow cytometry in D609-treated SKBr3 cells compared with untreated controls (mean ± SD values of three independent experiments). Click here for file

Additional file 5 **Induction of intracellular lipid bodies in MCF-7 cells following exposure to the PC-PLC inhibitor D609**. CLSM analyses (three-dimensional reconstruction images) of cells exposed to D609 (50 μg/mL) for the indicated time intervals, then fixed and stained with the lipid probe BODIPY 493/503 (green) for the detection of cytoplasmic lipid droplets and with phalloidin-633 (red) for monitoring morphological changes of actin cytoskeleton. Nuclei are reported in blue (DAPI). The corresponding control cell cultures are reported in the panels indicated by 'CTR' and 'CTR 120 h'. Scale bar, 20 μm. *Histogram on the bottom panel: *fold-increase of BODIPY 493/503 fluorescence intensity measured by flow cytometry in D609-treated MCF-7 cells compared with untreated controls (mean ± SD values of three independent experiments). Click here for file

Additional file 6 **Quantitative analysis of the migration and invasion potential of MDA-MB-231 cells**. The analysis was performed after staining with crystal violet the cells migrated to the lower side of the porous membranes, in the absence (migration assay) or in the presence (invasion assay) of Matrigel™. White columns: control cells; gray columns: D609-treated cells. **(A) **The number of both migrating and invading cells (calculated as the mean of cell counts evaluated in six 72.000 ⌠m^2 ^fields for each sample) significantly decreased when the transwell chamber invasion assay was performed in the presence of D609. **(B) **Images of the lower side of filters containing cells migrated in the absence or in the presence of Matrigel™. **(C-F) **The effect of D609 proved to be irreversible: the inhibition of migration **(C) **and **(E) **and invasion **(D, F) **was also observed when MDA-MB-231 cells were first treated with D609 (50 ⌠g/ml) for 24, 48 and 72 h, subsequently detached and seeded in the transwell chambers and allowed to migrate in absence of the inhibitor. All experiments were calculated in triplicate. Click here for file
