# Supplementary figures and images for: Inhibition of phosphatidylcholine-specific phospholipase C results in loss of mesenchymal traits in metastatic breast cancer cells
Source: Breast Cancer Res. 2012 Mar 19;14(2):R50. doi: 10.1186/bcr3151 (PMC3446384; doi:10.1186/bcr3151)

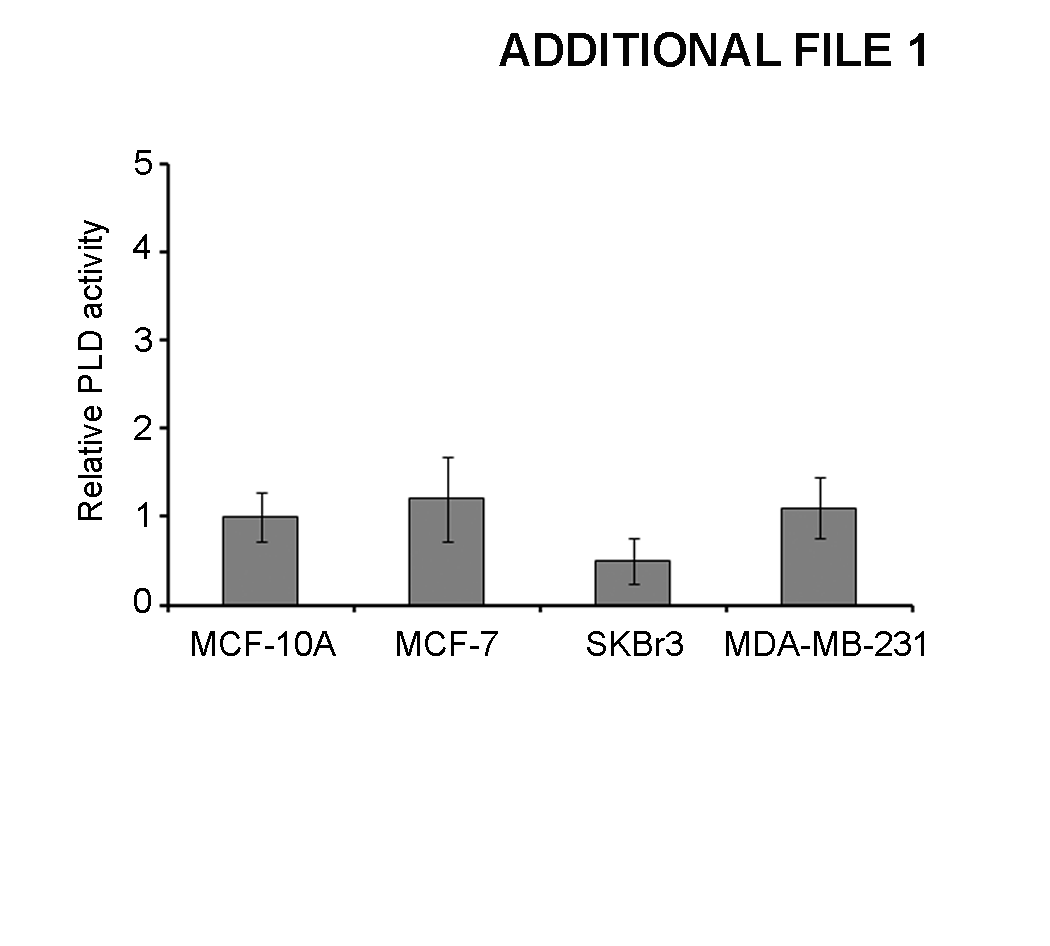

Supplement: Additional file 1 — Relative PC-specific phospholipase D (PLD) activity in breast cancer cells. PLD activity was measured by Amplex Red assay in breast cancer (BC) cell lines (MCF-7, SKBr3 and MDA-MB-231) compared with the human nontumoral mammary epithelial cells MCF-10A, all harvested at early confluence. Relative fold changes in BC cell PLD activity were normalized to the activity of MCF-10A cells, set to 1. Histograms represent the mean ± SD (n = 3). No significant differences were found in the relative rates of PLD in all investigated cell lines. [file bcr3151-S1.TIFF]

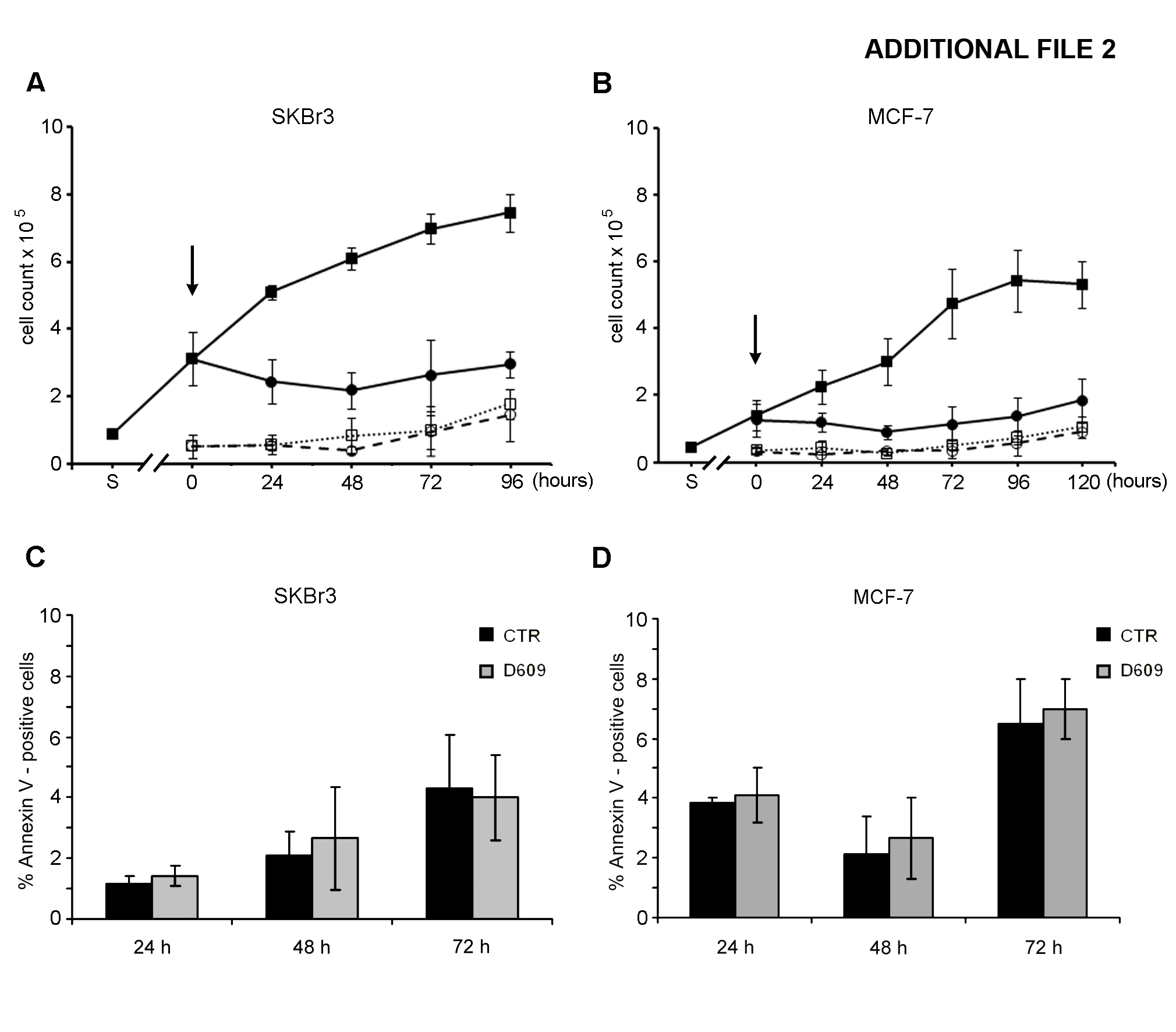

Supplement: Additional file 2 — Proliferation arrest induced by D609 (50 μm/mL) in SKBr3 and MCF-7 cells. (A) and (B) Cell counts (mean ± SD, n = 3) of SKBr3 and MCF-7 cells incubated in absence (black square, viable cells; white square, dead cells) or presence of D609 (black circle, viable cells; white circle, dead cells). Cells were seeded (S) 48 h before treatment, which started at t = 0 (arrow). (C) and (D) Percentages of Annexin V-positive cells in D609-treated and the respective CTR cells. [file bcr3151-S2.TIFF]

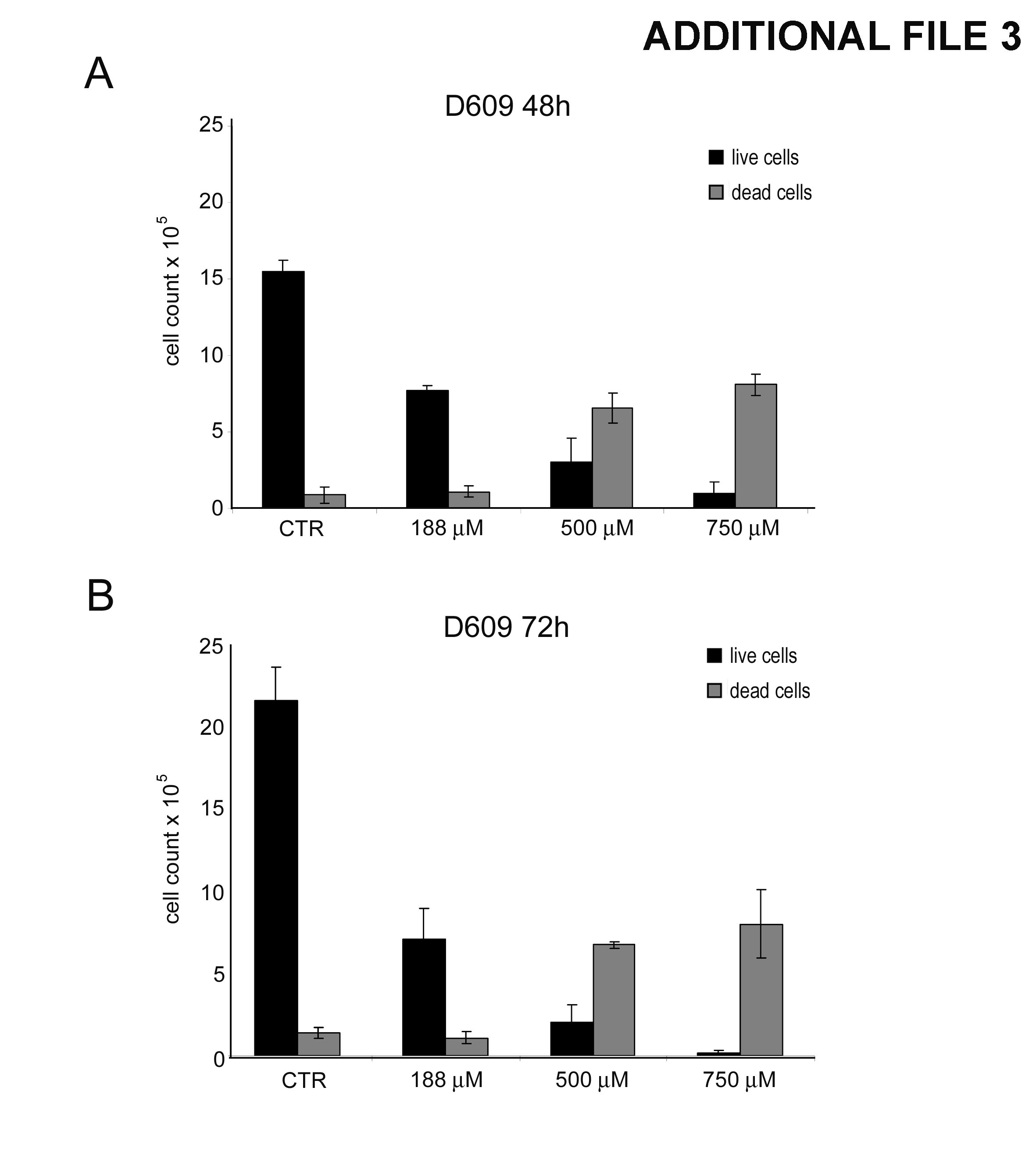

Supplement: Additional file 3 — Effects of different doses of D609 on cell growth and viability of MDA-MB-231 cells. Cells were incubated with different doses of D609 (188 μM, (corresponding to 50 μg/mL), 500 μM and 750 μM) for 48 h or 72 h. Cell proliferation was measured by cell count. Cell viability was assessed by trypan blue excluding test (experiments performed in triplicate). [file bcr3151-S3.TIFF]

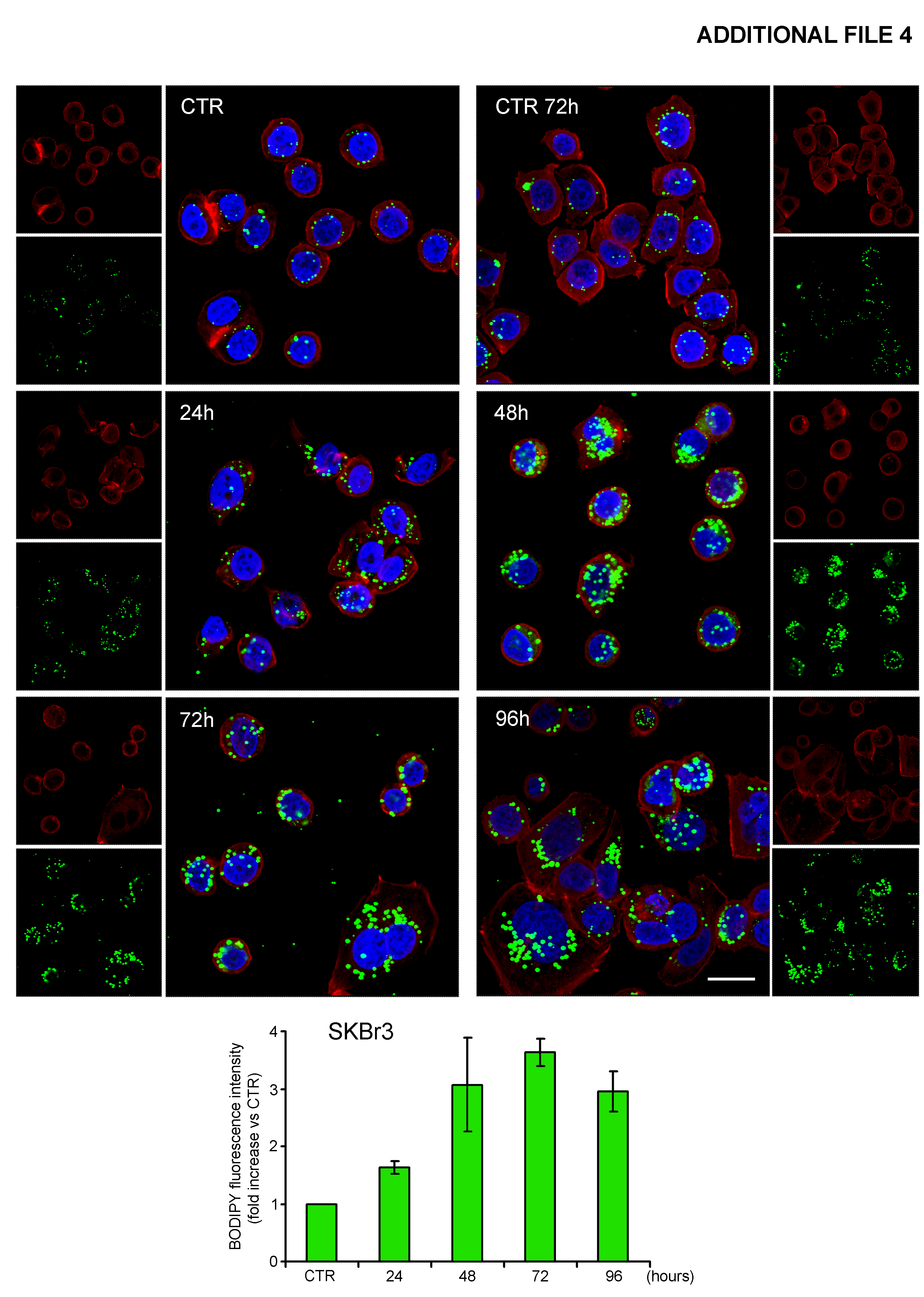

Supplement: Additional file 4 — Induction of intracellular lipid bodies in SKBr3 cells following exposure to the PC-PLC inhibitor D609. CLSM analyses (three-dimensional reconstruction images) of cells exposed to D609 (50 μg/mL) for the indicated time intervals, then fixed and stained with the lipid probe BODIPY 493/503 (green) for the detection of cytoplasmic lipid bodies and with phalloidin-633 (red) for monitoring morphological changes of actin cytoskeleton. Nuclei are reported in blue (DAPI). The corresponding control cell cultures are reported in the panels indicated by 'CTR' and 'CTR 72 h'. Scale bar, 20 μm. Histogram on the bottom panel: fold-increase of BODIPY 493/503 fluorescence intensity measured by flow cytometry in D609-treated SKBr3 cells compared with untreated controls (mean ± SD values of three independent experiments). [file bcr3151-S4.TIFF]

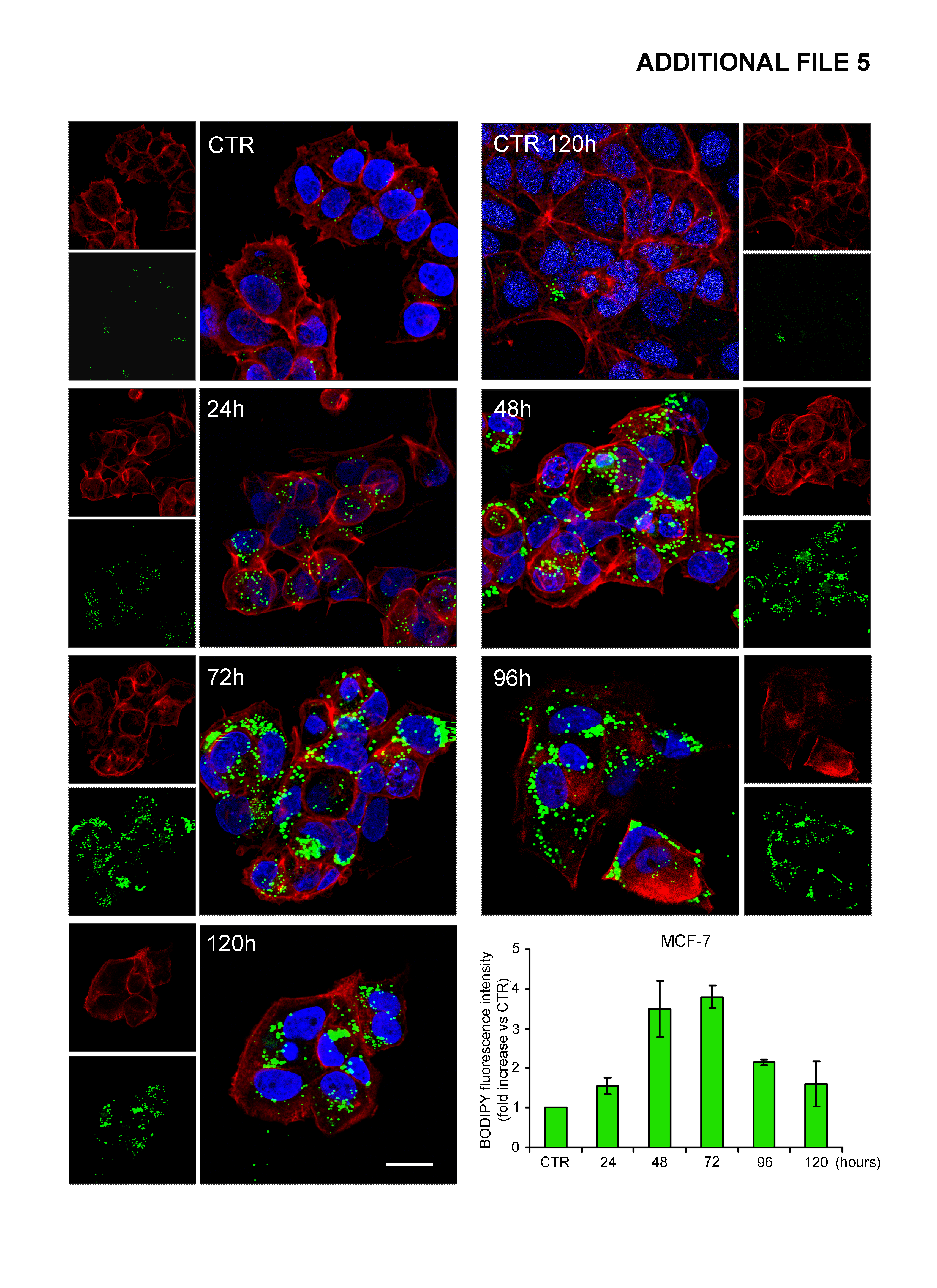

Supplement: Additional file 5 — Induction of intracellular lipid bodies in MCF-7 cells following exposure to the PC-PLC inhibitor D609. CLSM analyses (three-dimensional reconstruction images) of cells exposed to D609 (50 μg/mL) for the indicated time intervals, then fixed and stained with the lipid probe BODIPY 493/503 (green) for the detection of cytoplasmic lipid droplets and with phalloidin-633 (red) for monitoring morphological changes of actin cytoskeleton. Nuclei are reported in blue (DAPI). The corresponding control cell cultures are reported in the panels indicated by 'CTR' and 'CTR 120 h'. Scale bar, 20 μm. Histogram on the bottom panel: fold-increase of BODIPY 493/503 fluorescence intensity measured by flow cytometry in D609-treated MCF-7 cells compared with untreated controls (mean ± SD values of three independent experiments). [file bcr3151-S5.TIFF]

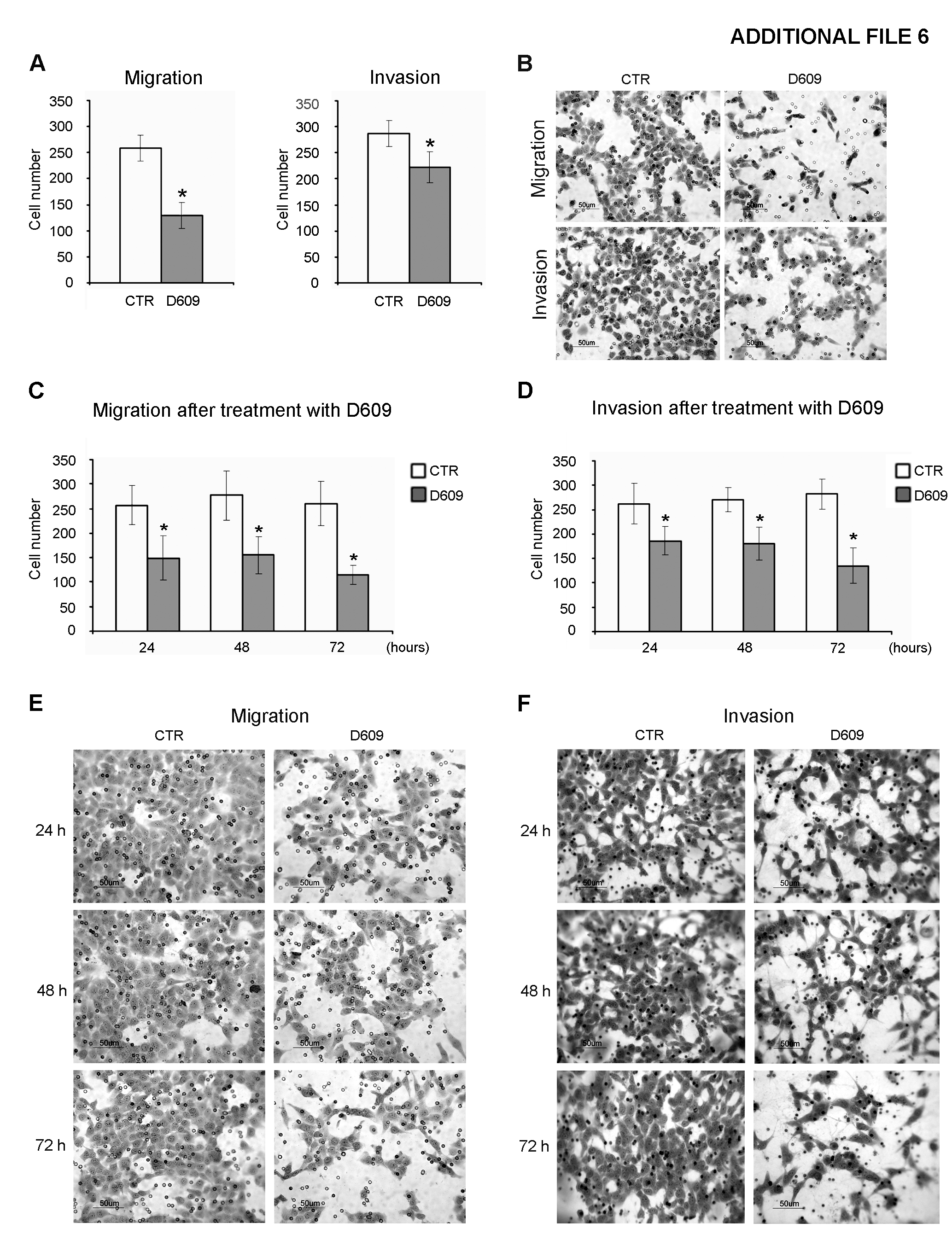

Supplement: Additional file 6 — Quantitative analysis of the migration and invasion potential of MDA-MB-231 cells. The analysis was performed after staining with crystal violet the cells migrated to the lower side of the porous membranes, in the absence (migration assay) or in the presence (invasion assay) of Matrigel™. White columns: control cells; gray columns: D609-treated cells. (A) The number of both migrating and invading cells (calculated as the mean of cell counts evaluated in six 72.000 ⌠m2 fields for each sample) significantly decreased when the transwell chamber invasion assay was performed in the presence of D609. (B) Images of the lower side of filters containing cells migrated in the absence or in the presence of Matrigel™. (C-F) The effect of D609 proved to be irreversible: the inhibition of migration (C) and (E) and invasion (D, F) was also observed when MDA-MB-231 cells were first treated with D609 (50 ⌠g/ml) for 24, 48 and 72 h, subsequently detached and seeded in the transwell chambers and allowed to migrate in absence of the inhibitor. All experiments were calculated in triplicate. [file bcr3151-S6.TIFF]
